# Boron Chemistry for Medical Applications

**DOI:** 10.3390/molecules25040828

**Published:** 2020-02-13

**Authors:** Fayaz Ali, Narayan S Hosmane, Yinghuai Zhu

**Affiliations:** 1 School of Pharmacy, Macau university of Science and Technology, Avenida Wai Long Taipa, Macau 999078, China; afayaz@must.edu.mo; 2 Department of Chemistry and Biochemistry, Northern Illinois University, DeKalb, IL 60115, USA

**Keywords:** boron chemistry, boron-containing compounds, boron cluster, carborane, boron neutron capture therapy, boron delivery agents for BNCT, medical applications

## Abstract

Boron compounds now have many applications in a number of fields, including Medicinal Chemistry. Although the uses of boron compounds in pharmacological science have been recognized several decades ago, surprisingly few are found in pharmaceutical drugs. The boron-containing compounds epitomize a new class for medicinal chemists to use in their drug designs. Carboranes are a class of organometallic compounds containing carbon (C), boron (B), and hydrogen (H) and are the most widely studied boron compounds in medicinal chemistry. Additionally, other boron-based compounds are of great interest, such as dodecaborate anions, metallacarboranes and metallaboranes. The boron neutron capture therapy (BNCT) has been utilized for cancer treatment from last decade, where chemotherapy and radiation have their own shortcomings. However, the improvement in the already existing (BPA and/or BSH) localized delivery agents or new tumor-targeted compounds are required before realizing the full clinical potential of BNCT. The work outlined in this short review addresses the advancements in boron containing compounds. Here, we have focused on the possible clinical implications of the new and improved boron-based biologically active compounds for BNCT that are reported to have in vivo and/or in vitro efficacy.

## 1. Introduction

Carbon chemistry has been widely studied over the past two centuries. Despite its neighbor in the periodic table, the study of boron chemistry is relatively a newcomer compared to the chemistry of carbon. However it is rich as building block of its own, and has been mostly used in dealing with carbon chemistry [1,2].

Boron is generally found in minute amounts in the human body (in an average individual it’s not more than 18 mg [3]). However, it has the potential to be considered as facilitator in new biological activities and can be utilized in pharmaceutical drug design. Mainly, the boron-containing bioactive molecules are of two types; one type of molecules contains a single boron atom, while the other is in the form of a boron cluster. Boron has the ability to instantly convert from a trigonal planar (sp^2^ hybridized) form, that is a neutral form, to a tetrahedral (sp^3^-hybridized) form, which is an anionic form in the single boron atom-containing compounds when used under physiological conditions. This provides the basis for using boron to design inhibitors for enzyme-catalyzed hydrolytic processes by adopting carbon-based transition states [4,5]. While the boron atoms as a whole in the cluster compounds are used rather than a separated or single boron atom, the unique interaction with targeted proteins are possible mainly due to their presence in cage structure [6,7,8].

In boron chemistry, the borane or boron hydride history started from the innovative work of Alfred Stock and his co-workers, who prepared a series of simple boranes [9]. However, the unusual nature of the bonding in borane became apparent in 1954 from the research of Lipscomb and co-workers describing the theoretical prediction in icosahedral borane [B_12_H_12_] [10]. The boron clusters concept was introduced by the research of Roberts and Longuet-Higgins suggesting that the stable form of icosahedron would have an overall 2-charge [11]. Hawthorne and Pitochelli validated this statement by reporting a series of synthetic investigations, including the formation and uses of decaborate [B_10_H_10_]^2-^ and dodecaborate [B_12_H_12_]^2-^ anionic species [2,12]. Similarly, a comparative analysis of the possibilities and characteristic features of the application of various polyhedral boron compounds, viz., the *closo*-decaborate anion [B_10_H_10_]^2–^, the *closo*-dodecaborate anion [B_12_H_12_]^2–^, the carba-*closo*-dodecaborate anion [CB_11_H_12_]^–^, carboranes C_2_B_10_H_12_, and the bis(dicarbollide) complexes [M(C_2_B_9_H_11_)_2_]^–^ (M = Fe, Co, or Ni), have been discussed by Sivaev et al., [13]. In addition, other scientist reported other boron-based compounds like an icosahedral closo B_12_^2-^ scaffold supporting multiple gadolinium complexes by Goswami et al., [14], and so on.

The actual motivation for the advancement of medicinal chemistry of boron was started from the use of boron neutron capture therapy (BNCT) for cancer treatment [15,16]. This is linked with the development in nuclear research technology and the availability of neutrons source suitable for the clinical treatment of cancers via BNCT. In 1960s, the discovery of polyhedral boron compounds facilitated the mission of BNCT through new boron carriers containing boron clusters rather than those with a single boron atom per molecule [17,18,19,20]. A major area of main group inorganic/organometallic chemistry has been developed from the study of electron-deficient boron clusters that overlaps with medicinal and organic chemistry to form a new area of bioorganic chemistry involving boron carriers for the treatment of tumor using BNCT model. Among new compounds of low-molecular weights for BNCT use are the carborane-containing carbohydrates, amino acids, nucleosides, nucleic acids and bases, lipids, DNA groove binders, and porphyrins [17,18,19,20]. Recently, a new generation of radiosensitizers was described for BNCT that includes biopolymers containing one or more carbonyl deposits. This class of boron species contains nucleic acids (DNA-oligonucleotides), carbonyl oligophosphates, and carbonyl peptides and proteins [5,19,21].

There are some strict requirements before using the boron drugs for BNCT application with regards to their toxicity, solubility and dosage accumulation in the cancer cells. However, the basic knowledge about pharmacokinetics and toxicity of boron compounds has been achieved by broad study of boron carriers for BNCT, that can be useful for the development of boron compounds for other biological applications.

Recently, new biological activities of boron cage molecules and their complexes have been revealed, including anticancer activity, anti-HIV activity, anti-rheumatoid arthritis activity, drug delivery and imaging for diagnosis and treatment of cancer and probing protein-biomolecular interactions [1,22,23,24].

These and other new observations clearly show that boron-containing compounds have great unexplored potential for medical applications and in bioorganic chemistry. It is not essential that boron should be the solution to all medical issues but it will be a welcome addition to the toolbox of medicinal chemistry. Three decades ago, fluorine was a newcomer to the medicinal chemistry and, today, the compounds of fluorine are synthesized on a routine basis in pharmaceutical research, and embrace a considerable part in the pharmaceutical market. The drug analogs bearing a variety of biological activities with a single boron atom or boron cluster molecules show that further exploration is required to realize the potential of boron in medicinal chemistry. Various reviews and books have been recently published showing the advances in boron chemistry and its applications [1,21,25,26,27,28,29,30,31,32,33,34,35,36,37], but this review will reveal many of the applications of boron chemistry in the medical field.

## 2. Boron Clusters for Medical Applications

Boron clusters are the main topic of several review articles and books [1,19,21,25,38], therefore, in this review only the basic information on boron clusters is provided; however, a little detail is given about their applications in medical field.

### 2.1. Structure Features of Boron Clusters

Carborane clusters and polyhedral boranes are characterized by delocalized electron deficient bonding, meaning that there are too few valence electrons for bonding to be described exclusively in terms of 2-center-2-electron (2c2e) pair bonds [39,40]. One characteristic of electron deficient structures is the aggregation of atoms to form 3-center-2-electron (3c2e) bonds, which typically results in the formation of trigonal faces and in hypercoordination [41]. The high connectivity of atoms in a cluster compensates for the relatively low electron density in skeletal bonds [41]. In drawing these polyhedral structures, it is common not to indicate the atoms at each vertex specifically. The typical boron and carborane clusters form the three-dimensional deltahedral shapes. Wade explained the clusters in their *closo* polyhedral forms, where n is the number of boron atoms containing n + 1 skeletal bonding electron pairs, typically resulting in anions of the type *closo*-[B_x_H_x_]^2-^ (x = 6−12 and above) [42].

The best-known types of polyhedral boron compounds which are most often used in medicinal chemistry are icosahedral dicarbadodecarboranes (C_2_B_10_H_12_), in which two CH units replaced the two BH vertices. Icosahedral carboranes have been known for more than half a century and are topologically symmetrical or globular molecules [25,43]. The molecular size of carboranes is almost larger than the volume of a rotated benzene ring and or adamantine, and the bonds of B–B and C–B in carboranes are of 12-vertex and are approximately 1.8 angstroms (Å) in length.

**Figure 1:**
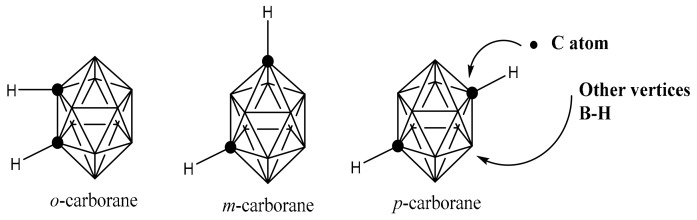
Structure of carborane due to the attachment of carbon at different sites.

Carboranes have a highly delocalized electron hydrophobic surface, and are reflected to be inorganic benzenes or three-dimensional aromatic compounds [44]. The carborane occupied almost 50% greater space than that of the rotating phenyl group. The carboranes are found in three isomeric forms due to the position of two carbon atoms within the cage as shown in Figure 1 The carborane system has the ability to enter in the substitution reaction at both boron and carbon atoms without degradation of the carborane cage. This is considered to be one of the most important features of this system for participation in various types of substitution reactions.

### 2.2. Properties of Boron Clusters for Medical Applications

Boron clusters have the following properties that are helpful in drug design: (a) the unique non-covalent interaction ability, including ionic interactions, σ-hole bonding and dihydrogen bond formation, is different from that of pure organic molecules due to its interaction with biological targets [21,44,45]; (b) ellipsoidal or spherical geometry and 3D arrangement which are helpful in the construction for 3D molecule; (c) the hydrophilicity, amphiphilicity, or lipophilicity allows the alteration of bioavaliabilty and pharmazcokinetics which is subject to the type of boron cluster utilized; (d) bioorthogonality, lessened vulnerability to metabolism and stability in a biological environment; (e) chemical stability and concurrent vulnerability to functionalization; (f) resistance to ionizing radiation, this property is essential for the radiopharmaceutical drug designing; and (g) high content of boron in the cluster is an important aspect to explore its application for BNCT [5,6,46].

The most widely studied compounds of boron in medicinal chemistry are carboranes, which are a class of cage-structured borons. In addition to carboranes, other boron clusters of dodecaborate anion and coordination compounds, accommodating different sizes and cage-structural topographies (metallacarboranes and metallaboranes), are also of great interest. Apart from these properties of desirable biological applications, the main advantage of boron cluster and their complexes is their abiotic nature and, consequently, they are biologically and chemically orthogonal to intuitive cellular constituents and resilient to catabolism.

### 2.3. Boron Cluster Implication for Drug Design

Specific and unspecific binding of boron clusters as protein ligands have been observed as explained in detail elsewhere [47,48]. In order to better understand the interaction modes of boron cluster compounds, these interactions should be carefully mapped, such as photoreactive groups [49]. Promiscuous binding is more frequently observed in fragment- based drug design, which is not desirable. However, it will be able to highlight molecular interactions and structural motifs capable of binding boron clusters.

The ability of boron clusters to influence the integrity and structure of a lipid membrane, and the strong binding of ionic boron clusters towards common cyclodextrins hydrophobic interior is an indication for the penetration of ionic boron clusters towards the hydrophobic environment of cell membranes. These properties are highly desirable for any type of drug design because boron clusters will not only convey water solubility to the compounds, but also allow them to penetrate the membrane. This behavior is required for a drug to reach to its target. Various studies were carried out to show the solubility of such compounds in a hydrophobic environment [50,51], thus Genady et al. [52] have recently demonstrated that a fluorescent dye can penetrate and accumulate in the cellular membrane of mammalian cells.

Computational methods, to handle boron clusters used in medicinal chemistry, have been explored by some studies as reported in the literature [53,54]. In organic structures, force fields have been developed for each functional group and are routinely applied in docking programs. However, no such field exists for boron and, particularly, for boron clusters. Therefore, the carbon force fields were used to treat the boron cluster (almost as an adamantane) to obtain the data in the literature [53,54]. The computational outcomes obtained from imitating boron clusters with carbon force fields must be considered fairly unreliable. The properties of the boron cluster are vastly different in comparison to adamantanes or carboxylic acid, which are shown by the lack of activity of adamantane-substituted *m*-, and *p*- carbonyl derivative when compared to *o*-derivative of indomethacin [55], or the huge difference in nicotinamide phosphoryltransferase inhibitors comprising either a neutral carborane or an adamantane [56]. Therefore, appropriate force fields need to be developed for ionic and neutral boron clusters before incorporating them in docking programs.

## 3. Boron Neutron Capture Therapy (BNCT)

### 3.1. Mechanism of BNCT

**Figure 2:**
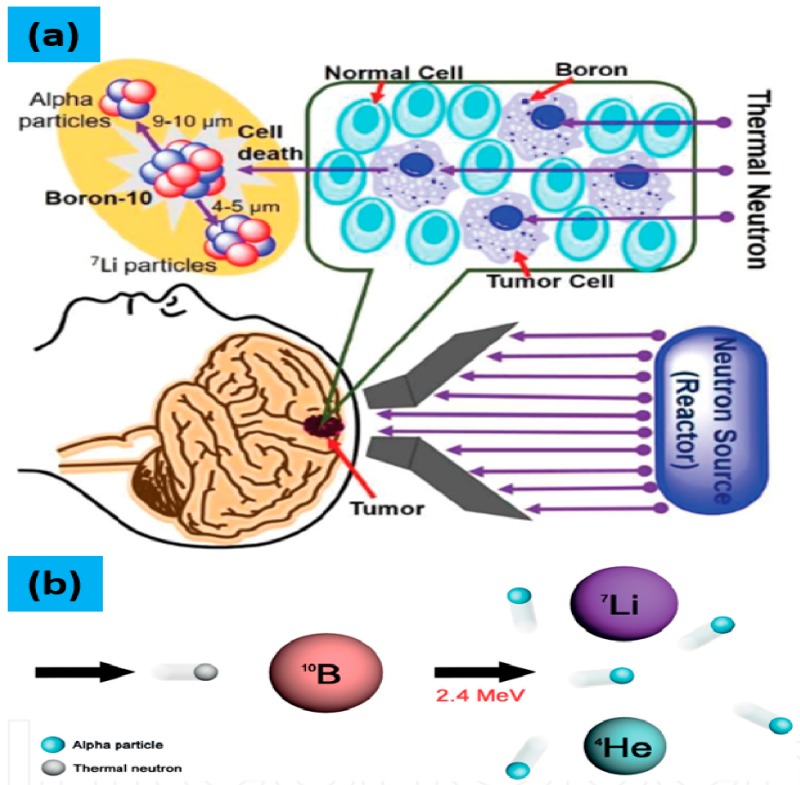
(**a**) Graphical depiction of the principle of BNCT, that show how localized boron particles within the tumor can be bombarded with low energy thermal neutrons to produce alpha particles (Reprint from Ref. [61]). (**b**) BNCT reaction scheme, where the thermal neutrons were bombarded on ^10^B resulting in a nuclear reaction releasing α particles and ^7^Li nuclei within a tumor cell, offering a localized lethal effect.

Recently, boron neutron capture therapy (BNCT) has attracted attention because it is a strategy for binary targeting and noninvasive treatment of cancer. BNCT is a possible treatment methodology for cutaneous melanomas, extramammary Paget’s diseases of genital regions, vulvar melanoma, neck and head cancers, and high-grade gliomas [30,57,58,59,60]. An appropriate number of ^10^B atoms must be introduced to the neoplastic cells that could be irradiated with adequate number of neutrons to obtain a successful BNCT reaction (the graphical representation of BNCT is shown in Figure 2). It is reported that a cell required an average of 3–7 alpha-particles (from ^7^Li nuclei) and ^10^B/g to destroy a tumor tissue of ca. 15 µg [19]. Therefore, the production of clusters having high boron content play an important role if BNCT is clinically accepted to evolve the treatment for cancer. In the early days of formulating boron carriers for BNCT, mostly mixture of ^10^B (19.9%) and ^11^B (80.1%) isotopes of natural boron were used. In clinical applications, the ^10^B enriched boron carriers are used due to its higher neutron capture cross-section 3837 barns as compared to 0.005 barns of ^11^B. Alpha particles are more valuable than X-ray for radiotherapy, due to their following properties (a) no need of oxygen for alpha particles to increase their biological effectiveness; [59], (b) much higher relative biological effect (RBE) of alpha particles [36], and (c) Both dividing and nondividing tumor cells can be killed by alpha particles [8]. These properties enable alpha particles tagged with B-10 to selectively kill various types of cancer cells without damaging the normal cells, that helps to prevent the side effects for patients.

The ideal boron-containing agent should fulfill the following requirements: (i) low systemic toxicity; (ii) approximately 30 μg 10 B/g tumor concentrations; (iii) high tumor uptake and low normal tissue uptake; and (iv) rapid normal tissue clearance but persistence in tumor tissue during BNCT.

### 3.2. Current BNCT Agents

**Figure 3:**
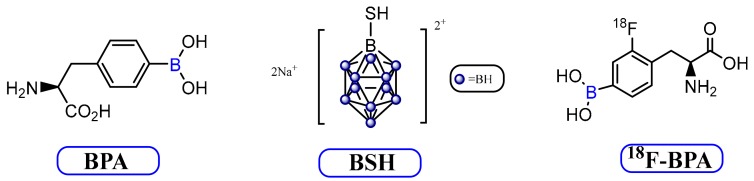
Structures of BPA, BSH and ^18^F-BPA.

In order for BNCT to be successful, it must get enough boron to the tumor cell [8]. Two types of boron-containing drugs have been utilized for clinical treatment for more than 1000 patients using BNCT. These are mercaptoundecahydrodecaborane (BSH) and boronophenylalanine (BPA), whose structures are illustrated in Figure 3 [7]. However, the ideal dosage of either BPA or BSH or the combination of the two in the delivery system to the patients with high grade gliomas has yet to be established. The Swedish group reported [62,63], that by increasing the duration of the infusion time for BPA dose would be a good starting point, but microdistribution and improving tumor uptake could require more than this strategy. Therefore, BNCT for additional clinical applications is somewhat obstructed due to poor boron transfer competence of BSH and BPA [1,35,64]. Nonetheless, an optimal response to BNCT for improving tumor uptake of boronated compounds critically depends on performing the neutron irradiation at the time of maximum boron accumulation (or highest T/N ratio). Determining the maximum boron accumulation in patients’ tumor, reliably, is one of the biggest limitations of BNCT, especially considering the fact that inconsistency always exists in different kinds of tumors among patients. Positron emission tomography (PET) guiding BNCT can potentially overcome this challenge by using a dual modality agent [65] in which the real-time boron accumulation within the tumor of the patients can be monitored. The 4-borono-2-^18^F-fluorophenylalanine (^18^F-BPA) is one of the examples of a dual modality BNCT agent, which is a radiolabeled derivative of BPA as shown in Figure 3 The ^18^F-BPA uptake for head and neck cancers can be correlated with the uptake of ^18^F-fluorodeoxyglucose [66]. The ^18^F-BPA administration has also been reported for numerous types of tumors such as malignant melanomas, malignant gliomas and various head and neck cancers with tumor/normal tissue ratios ranging from 1.5 to 7.8 [67,68].

### 3.3. Development of Novel BNCT Agents

Recently, many small molecule-based boron carriers have been explored in the preclinical trials, some of which are discussed here. However, due to their insufficient accumulation of boron in tumor cells, clinical studies have been limited [34,46].

Researchers tried to overcome this problem by developing boron-containing nanoparticles for delivering at least 20 ppm of boron to the tumor cells [61,69]. Some of the developed boron-containing nanoparticles have significantly improved the delivery efficacy of boron to the tumor cells and exhibited excellent tumor destruction in animal models [61,70]. Nevertheless, boron-containing nanoparticles shows in vivo toxicity i.e., resistance to degradation, which is the main concern for its clinical translation [71,72,73]. To overcome these problems, some researchers have shown interest in preparing biodegradable boron-containing nanomedicines with the help of biodegradable polymers. This includes natural polymers such as chitosan, gelatin, albumin, collagen and alginate [74,75,76], or synthetic polymers such as poly-(ethylene glycol)-block-poly(lactide) copolymer (PEG-b-PLA), poly(D.L-lactide-co-glycolide) (PLGA), and polylactides (PLA) as coating layers [77,78,79,80], and boron compounds as boron carriers [61,81,82,83]. These polymers undergo hydrolysis in a physiological environment due to their polyester structure. Compared to the natural degradation mentioned above, the exogenous response is more precise and controllable [84,85,86], compared to the on-demand decomposition process [46]. A third generation of boron-containing compounds were recently investigated. A stable boron cluster is attached to a tumor-targeting moiety via a hydrolytically stable linkage, such as low or high molecular weight agents [87]. Boron-containing amino acids, biochemical precursors, polyhedral boranes, DNA-binding agents, mannose, glucose, ribose, galactose, fucose, lactose molecules, amines, benzamides, nitroimidazole, nicotinamides, phosphates, phosphonates, isocyanates, azulenes, phenylureas, thioureas, and deualinium derivates are included in low molecular weight agents while high molecular weight agents include liposomes, receptor-targeting agents, and monoclonal antibodies. These new generation boron-containing agents show better selective targeting properties when compared to the old generation boron compounds [87]. However, their biological properties depend on the density of the targeted sites and very little data have been reported to date on the third-generation of boron-containing agents. Some of these newly reported boron-containing agents are discussed below.

#### 3.3.1. Boron Nanoparticles with BNCT

Due to significant boron content in the pure boron nanoparticles, they are considered as potentially promising boron carrier agents. Although they can be prepared by various synthetic methods, the commonly employed techniques are pyrolysis, chemical vapor deposition (CVD), thermal plasma, reduction in solution, ball milling and arc discharge [29,88,89,90]. Icten et al. reported [91] the use of the ball milling method for the preparation of magnetic dopamine-functionalized boron nanoparticles, which show a size range of 100–700 nm. As a result, the ball milling method produces, in one step, both boron nanoparticles and their functionalization [91]. This group further investigated the synthesis of magnetic nanocomposites containing polyethylene glycol, Fe_3_O_4_ and mono or bis(ascorbatoborate) [92]. The resulting nanocomposites had an average size of 10–15 nm and show good paramagnetic behavior at 300 K [92]. These materials are considered to be the potential constituents for magnetic biomedicine, since the composites combined with ascorbic acid are recognized as useful antitumor and radical scavenging agents.

**Figure 4:**
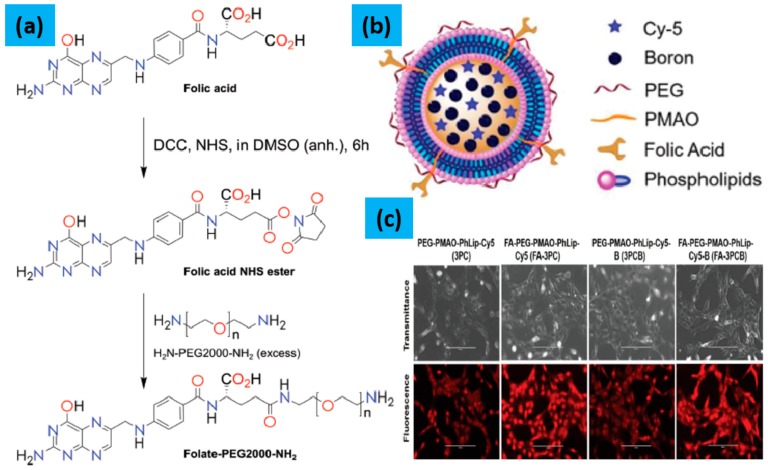
Schematic image of boron encapsulated liposome (Adopted from Ref. [61]).

Recently, Sing et al. [61], reported the synthesis of pure boron nanoparticles comprised of a liposome of azolectin-based phospholipid using the water-in-oil emulsion method. This new material contains polyethylene glycol (PEG) and poly(maleic anhydride-alt-1-octadecene) (PMAO) on the surface, and boron nanoparticles and Cy5 near infrared (NIR) fluorescent dye in the core (3PCB) as shown in Figure 4, which is considered as an alternative BNCT agent. For improvement in accumulation and targeted delivery of boron to cancer cells, a tumor-specific targeting ligand, folic acid (FA), was conjugated to PEG to produce a folate-functionalized liposome (FA-3PCB). The liposomes exhibited a zeta potential of —38.0 ± 1.5 mV and an average diameter of 100–120 nm. The targeting capability of FA-conjugated liposomes was confirmed by monitoring the cellular uptake by fluorescence microscopy. It was observed that the accumulation of FA-conjugated liposomes in C6-brain tumor cells was much higher than that of non-FA conjugated liposomes under the same conditions. The quantification of sufficient accumulation of boron in cancer cells for therapeutic benefit from BNCT was confirmed by Inductively Coupled Plasma Mass Spectrometry (ICP-MS). These liposomes show blood-brain barrier (BBB) crossing ability, excellent stability, and low cytotoxicity under physiological conditions. Thus, these liposomes are considered to be promising new boron carriers for BNCT.

#### 3.3.2. Boron Nitride Nanotubes/Nanoparticles with BNCT

Recently, highly water dispersible boron nitride (^10^BN) has been synthesized by using the solvothermal method at relatively low temperature [93]. The morphological structural analysis of ^10^BN has been carried out along with a cytotoxicity analysis on normal (HEK-293) and cancer (HeLa, MCF-7) cells, the results illustrates that ^10^BN is relatively less toxic and produces insignificant oxygen species. The promising BNCT antitumor effect was observed in ^10^BN treated HeLa cells. The thermal neutron fluence of ~6.3× 10^12^/cm^2^ resulted in almost 50% cell killing of BN treated HeLa cells. Another study reported the antitumor effects of boron nitride nanotube (BNNTs) with BNCT towards melanoma cells [94]. The accumulation of BNNTs in B16-melanoma cell was three times higher than BSH and their antitumor effect with BNCT was also observed higher than BSH. The BNNTs are also attracting the attention of the scientific community as did the carbon nanotubes (CNTs) earlier. Nonetheless, the BNNTs are lightweight with excellent mechanical properties and offer strong resistance to oxidation when compared to CNTs. Therefore, the BNNTs used in nanocomposites are considered to be multifunctional materials with promising applications in biomedical fields, nanoscale electrical devices, optical systems and in space radiation shielding [95,96,97,98]. In addition, the high boron density of BNNTs make them to be possible aspirants to the boron agents [94,99,100,101].

**Figure 5:**
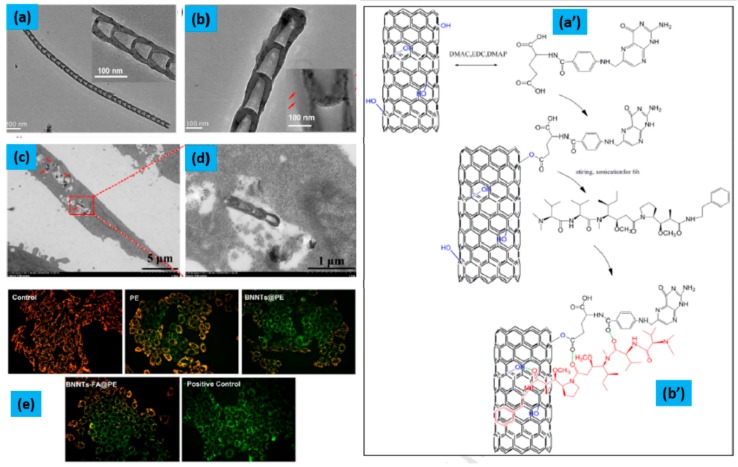
(**a,b**) TEM images of BNNTs-OH and BNNTs-FA@PE, respectively (the insert is the enlarged TEM images); (**a’,b’**) represents the BNNT and the conjugation of FA and PE on the surface of a BNNT, respectively; (**c,d**) TEM images of Hep G2 cells incubated with BNNTs-FA for 4 h of Low magnification and high-magnification, respectively; (**e**) the mitochondrial membrane potential (ΔΨ) of control, PE, BNNTs@PE, BNNTs-FA@PE and positive control group, and carbonyl cyanide *m*-chlorophenylhydrazone was used to conduct positive control (reprint from Ref. [102]).

In 2019, Wei et al. [102] reported the boron nitride nanotubes-conjugated folate (BNNTs-FA)-based targeted drug delivery strategy to improve the efficacy of Auristatin PE (PE). The PE as an anti-microtubule agent possesses good anticancer activity. However, strong side effects and poor targeting ability limits its clinical applications. Therefore, the targeted delivery of PE may overcome the disadvantages associated with PE, being very conducive to continuing clinical trials of PE. As pointed out above, BNNTs have attracted considerable attention for drug delivery due to their unique physical and chemical properties. Wei [102] found that PE was successfully loaded via π-π stacking and hydrogen bonding interactions onto BNNTs-FA and due to the increased cellular uptake of PE, mediated by the FA receptor, the BNNTs-FA@PE exhibited stronger cytotoxicity to Hep G2 cells than free PE and BNNTs@PE complexes, as shown in Figure 5 Also, BNNTs-FA@PE demonstrated excellent antiproliferative activities in time- and dose-dependent manners. Additionally, the BNNTs-FA@PE induced apoptosis of Hep G2 cells by reducing the mitochondrial membrane potential via intrinsic mitochondria–mediated pathway, activating Caspase-9 and Caspase-3. This study, demonstrates the construction of the BNNTs-FA@PE system, that significantly improves the PE effect on the target and may be considered as a promising material for the treatment of liver cancer in the near future.

**Figure 6:**
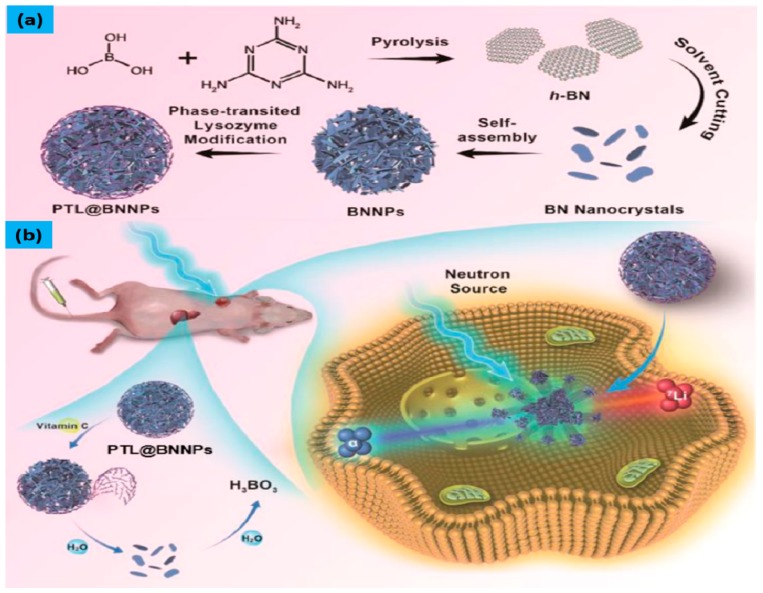
(**a**) Schematic representation of the synthesis of the PTL@BNNPs, (**b**) Illustration of PTL@BNNPs Based BNCT and its degradation (Reprinted from Ref. [46]).

Recently, it has been reported that boron nitride nanoparticles (BNNPs) exhibited good results in treating triple negative breast cancer in mice [46]. Since BNNPs show good biocompatibility and have high boron content capacity, they are considered as promising boron carriers. On-demand degradable boron carriers were designed and were coated by a phase-transitioned lysozyme (PTL) as shown in Figure 6a. The PTL protects the BNNPs from hydrolysis during blood circulation and can be readily removed by vitamin C after NCT. The report also indicated that the coated BNNPs showed high boron accumulation in the tumor while maintaining its good ratio between tumor and nontumor cells. After neutron irradiation, the tail-vein injections of vitamin C were followed, and it was found that BNNPs were rapidly cleared from major organs according to ex vivo ICP-OES analysis (Figure 6b). Compared with the control group, animals treated with BNCT showed suppression of tumor growth, while almost negligible side effects were observed. This strategy not only utilized the high boron content of BNNPs but also successfully performed an on-demand degradation of BNNPs to avoid the potential toxicity caused by the long-term accumulation of nanoparticles. Even high accumulation of B atoms inside the cells have been shown by these nanoparticles but they have failure in the therapeutic window for BNCT. More improvements are still needed to design a ^10^B-enriched BNNPs source [103,104].

#### 3.3.3. Gold Nanocluster/Nanoparticles with Borane for BNCT

**Figure 7:**
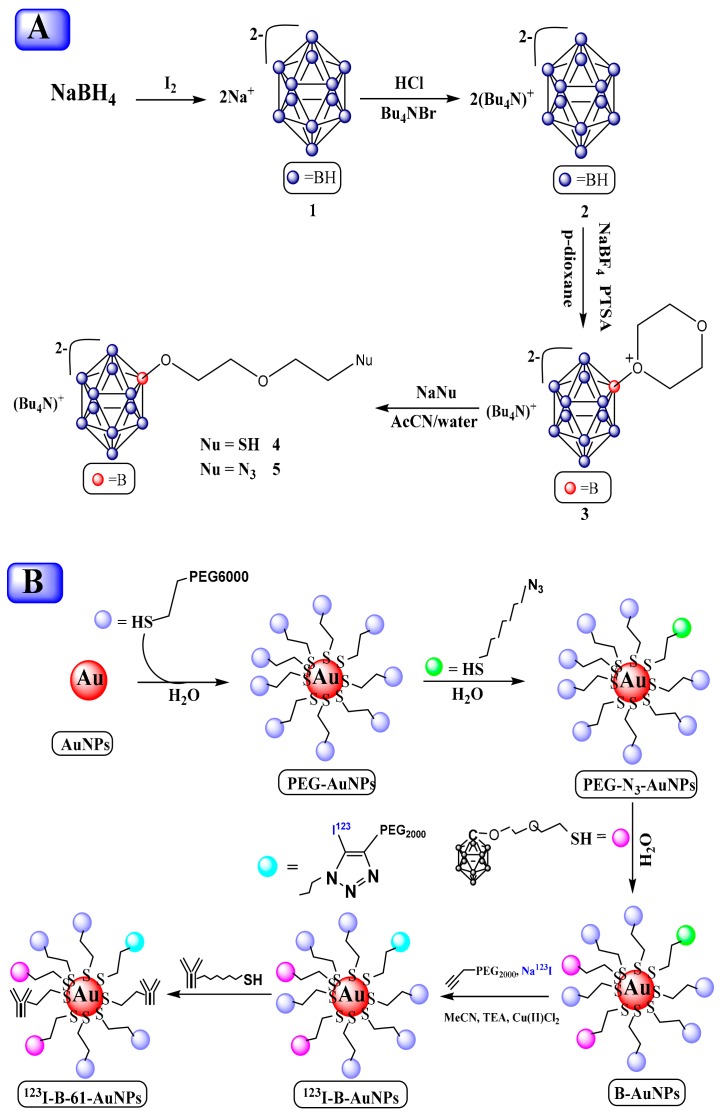
(**A**) The synthetic scheme of boron cage-SH (4), (**B**) The synthetic scheme of B-AuNP_s_, ^123^I-B-AuNP_s_ and ^123^I-61-B-AuNPs.

Recently, gold nanoparticles (AuNPs) have been utilized as delivery agents for releasing selective cytotoxic payloads to the tumor. However, studies demonstrating the in vivo distribution or pharmacokinetics of boron-containing AuNPs via noninvasive imaging are lacking. Chun *et. al.* [105] have used theranostic AuNPs with boroncage assemblies (B-AuNPs) to estimate its practicality for BNCT application. They subjected the commercial citrate-coated AuNPs to PEGylation, azide addition, and carborane modification on the surface. Further, they conjugated anti-HER2 antibody (61 IgG) with boron-containing PEGylated AuNPs to form 61-BAuNPs. The anti-HER2 antibody (61 IgG), provided by Prof. An-Suei Yang (Academia Sinica, Taiwan), and boron cage-SH (BC-EG-SH; Figure 7A), courtesy of Prof. Ming-Hua Hsu, and the surface modification of AuNPs are shown in Figure 7B. The radio thin-layer chromatography (radio TLC) was used to determine the radiolabeling efficiency and dynamic light scattering (DLS) of boron-containing AuNPs. The pharmacokinetics of radioiodinated AuNPs in N87 gastric cancer xenografts was determined by computed tomography (CT)/noninvasive single-photon emission computed tomography (SPECT) imaging, and boron contents in muscle and tumors were determined by inductive coupled plasma mass spectrometry (ICP-MS). It was observed that the diameter of B-AuNPs increased by approximately 25 nm after the 3-step modification, and the diameter of AuNPs was not affected by antibody conjugation. The radioactive iodine (I^123^) in AuNPs under copper catalysis was introduced with the help of Click chemistry. The radiolabeling efficiency of ^123^I-B-AuNPs and ^123^I-61-B-AuNPs was observed upto 60 ± 5% and the radiochemical purity (RCP) of these NPs after purification was greater than 90%.

It was reported that the micro CT/SPECT imaging exhibited the tumor-to-muscle (T/M) ratio of ^123^I-B-AuNP-injected mice reached 1.91 ± 0.17 at 12 h post-injection, while that of ^123^I-61-B-AuNP-injected mice was 12.02 ± 0.94. Therefore, it was indicated that the increased uptake of AuNPs by the thyroid at 36 h after the administration of ^123^I-61-B-AuNPs was observed due to antibody-mediated phagocytosis. They also reported that the T/M ratio, assessed by ICP-MS, of mice injected by B-AuNP- and 61-B-AuNP was 4.91 ± 2.75 and 41.05 ± 11.15, respectively. The results show the successfully developed detectable HER2-targeting boron-containing AuNPs with high RCP and an acceptable yield. From this study, it may be considered that noninvasive imaging could be a valuable tool for the noninvasive determination of the pharmacokinetics of AuNPs and measurement of boron concentration in the tumor.

Wang et al. [106] reported the gold nanoclusters with carborane amino derivatives for the targeted delivery of the carborane compound to the tumors and precise bioimaging of cancer cells. They prepared carborane derivative (NH_2_NH_3_)^+^[7-NH_2_(CH_2_)_3_S- 7,8-C_2_B_9_H_11_]^-^ according to the literature [107], and stored it at —18 °C. Gold nanoclusters (GNCs) were freshly prepared and dispersed in PBS (pH 7.2). Fluorescent GNCs were produced using glutathione (GSH), according to the reported method in the literature [108]. The accurate tumor imaging by EPR effect and long-term accumulation in tumor cell by nanometer size effect have been observed. This strategy is good for attaining the accurate position of tumor cells with carborane derivatives and thus decreases the chances of normal tissue damage. In addition, it facilitates the real-time fluorescent visualization to monitor delivery process of the carborane to the targeted tumor, thus endorsing the effect of BNCT treatment through imaging guided therapy.

**Figure 8:**
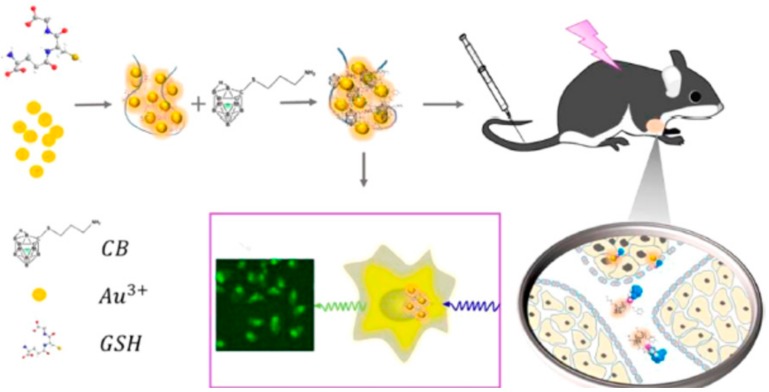
Illustration of the bioimaging process for cancer cells using gold nanocluster with carborane derivative (reprint from Ref. [106]).

The schematic illustration of this study is shown in Figure 8 In another study, the gold NPs were stabilized with polyethylene glycol (PEG), functionalized with bis(dicarbollide), radiolabeled with ^124^I at the core and the shell, and examined in vivo with a mouse model by using positron emission tomography (PET). The results showed a poor accumulation in tumor and major accumulation in liver, lungs and spleen suggesting that the tuning the size and geometry of the gold core is essential [109].

#### 3.3.4. Boron-Based Amino Acids for BNCT

**Figure 9:**
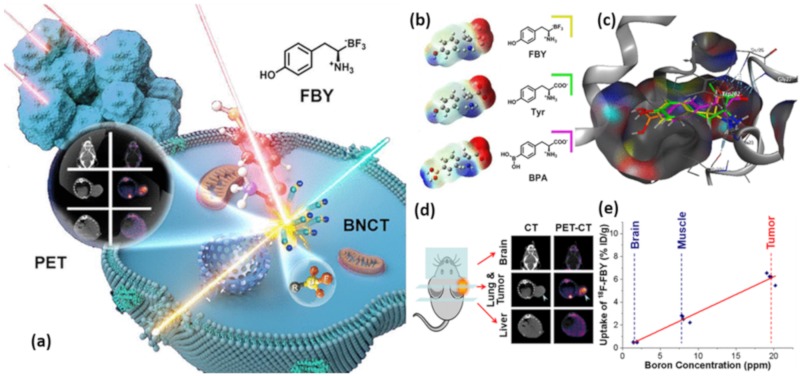
(**a**) The schematic presentation of the method reported by Ref. [111]. (**b,c**) The computational studies of FBY, Tyrosine, and BPA. (**d**) CT and PET-CT images of the brain, lung tumor and liver retention of [^18^F]FBY. (**e**) Correlation between standard uptake value (SUV) of organs and boron concentration of [^18^F] FBY. (adopted from Ref. [111]).

Most of the amino acids utilized in BNCT for precise treatment of malignant brain tumors [110], have been reported and the review published recently by Jiyuan et al. [111] reported the development of a metabolically stable boron-derived tyrosine (expressed as fluoroboronotyrosine, FBY) as a theranostic agent for both boron delivery and cancer diagnosis, leading to PET imaging-guided BNCT in the treatment of cancer as shown in Figure 9a. The computational study of FBY, Tyrosine, and BPA showed similarities between them to a greater extent. The FBY, Tyrosine, and BPA chemical structures and molecular electrostatic potential (MEP) images were shown in Figure 9b, where red indicates the distribution of negative charge and blue indicates the distribution of positive charge. The Figure 9c exhibited LAT-1 (gray) in solid ribbon representation, the predicted structure of the LAT-1 and FBY (yellow)/Tyrosine (green)/BPA (pink) complex, LAT-1. The hydrogen bonds between LAT-1 and ligands are known to be highly conservative involving residues Trp202, Ser26, Gly27, Ile205, and Ile23 which are shown as dotted sky-blue lines. The [^18^F]FBY was synthesized in high radiochemical purity (98%) with high radiochemical yield (50%).

It was also demonstrated that the prepared FBY showed high similarity with natural tyrosine. The uptake of FBY in murine melanoma B16-F10 cells was L-type amino acid transporter (LAT-1) dependent and reached up to 128 μg/10^6^ cells. While the FBY displayed high stability in PBS solution, the [^18^F]FBY PET showed up to 6% ID/g in B16-F10 tumor and notably low normal tissue uptake (tumor/muscle = 3.16 ± 0.48; tumor/blood = 3.13 ± 0.50; tumor/brain = 14.25 ± 1.54). Moreover, the administration of [^18^F]FBY tracer along with a therapeutic dose of FBY showed high accumulation in B16-F10 tumor and low normal tissue uptake.

The FBY enriched tumor having more than 20 ppm of boron and a desired correlation was established between tissue boron concentration and uptakes from PET imaging. The PET images, shown in Figure 9d, were recorded nearly 75 min after injection of 20 mg of FBY and [^18^F]FBY, intravenously. While the CT and PET-CT images of the brain coronal showing the [^18^F]FBY uptake, the representative transverse CT and PET-CT images of lung tumor and liver showed prominent [^18^F]FBY uptake in the B16-F10 lung tumor and the increased liver retention of [^18^F]FBY, respectively. The correlation between PET-image and boron biodistribution was established (Figure 9e) indicating the possibility of estimating boron concentration via a noninvasive approach. Using thermal neutron irradiation, B16-F10 tumor-bearing mice injected with FBY showed significantly prolonged median survival without exhibiting obvious systemic toxicity. In conclusion, FBY holds great potential as an efficient theranostic agent for imaging-guided BNCT by offering a possible solution of measuring local boron concentration through PET imaging and can be used for clinical trials.

#### 3.3.5. Boron-based Polymers for BNCT

A review, published recently by Chauhan et al. [112], covered the syntheses of most of the boron-based polymers and their application in BNCT treatment. This review discusses a few boron-based polymers that could be useful for biomedical applications. Ochiai et al. [113] reported polymer composites based on boron-doped diamond powder (BDDP), which have been utilized for the treatment of dental problems. The BDDP-based polymer was considered to be the practical species, since it possesses qualities, such as hard to crack or peel, and appreciable durability even with repeated bending during electrolysis. Chen et al. [114] proposed the use of iRGD-modified polymeric nanoparticles for active targeted delivery of boron and doxorubicin (DOX) in BNCT. They covalently grafted the ^10^B-enriched BSH by PEG-PCCL for the prearation of ^10^B-polymer and then modified its surface by iRGD, followed by incorporation of DOX into polymers, physically. The resulting polymers show enhanced accumulation of ^10^B in tumors when compared to BSH and prolonged blood circulation, along with the favorable boron concentration ratios for tumor:normal tissue (tumor:muscle = 19.49, tumor:blood = 14.11) in A549 tumor-bearing mice after 24 h of injection. The highest tumor accumulation of DOX was confirmed from both quantitative measurement and fluorescence imaging at 24 hrs after injecting iRGD-modified polymers [114]. However, more future studies are required to utilize these types of polymers for clinical trials. The properties, such as electrical carrier injection, transport, photoluminescence, solid-state luminescent, and reflective index make such boron-based polymers useful for molecular machines, multiphoton microscopy, and waveguides, optical data storage, cell biology, and tumor hypoxia (Table 1).

## 4. Accelerator-Based BNCT (AB-BNCT)

The reactor-based BNCT facilities are now shifting to accelerator-based BNCT (AB-BNCT). Consequently, various types of AB-BNCT have been planned and developed. Currently, only Japan uses these facilities for treatment purposes. However, in some other countries several new projects have been proposed and the new BNCT facilities are under construction, including those in Finland, China, Taiwan and Argentina [115,116]. Recently, Hirose et al. [116] reported the initial results of the phase-II trial of AB-BNCT by using cyclotron-based neutron generator and ^10^BPA in patients with recurrent and locally advanced head and neck cancer. The 8-recurrent squamous cell carcinoma (R-HNSCC) and 13 recurrent/locally advanced non-squamous cell carcinoma (R/LA-HNNSCC) patients were enrolled and received AB-BNCT. The tumor response was assessed every 4 weeks for the first 3 months. By the first 3-months, central review of the local control was observed in 20 patients out of total 21 patients. Except for asymptomatic hypermylasemia associated with acute parotitis, all the patients had no ≥ grade 4 toxicity. This data is still immature and needs further exploration to know the treatment efficacy [116]. However, it can be suggested that AB-BNCT could provide a promising treatment option for the R-HNSCC and R/LA-HNNSCC patients. An issue with AB-BNCT is the targets and it required more time to obtain information regarding its lifetime and stability. Low cost of construction and running, as well as high stability, are required for medical practice. Therefore, for future AB-BNCT application the more suitable accelerator development is still desirable.

## 5. Treatment of Different Cancer Tumor with BNCT

### 5.1. Recurrent Head and Neck Regional Tumor Treatment with BNCT

The treatment of recurrent tumors of the head and neck (HN) region by using BNCT have been applied to the second largest group of patients who had no other treatment options or reached normal tissue tolerance level. Although the number of patients treated in Taiwan, Finland and Japan by this method are relatively small, the result of this treatment shows some very remarkable clinical consequences [117,118,119,120].

Wang et al. [121] reported that a total number of 17 patients with recurrent HN tumors were treated with two doses of BNCT over 28 day intervals using BPA-F as the boron delivery agent. Although the toxicity was acceptable and the response rate was high (12 out of 17 patients), the tumor recurrence was common near or within the treatment site. Similar results were observed by Finnish and Japanese clinicians, who also treated the recurrent HN tumor patients. The resulting recurrence after treatment with BNCT might be due to the poor microdistribution of BPA-F in some region of the tumor, such as non-homogenous uptake of BPA-F by the tumor cells [30,122].

While the “best” boron delivery agent are yet to be developed, the optimal dose and delivery of BPA (alone or in grouping with BSH) is the best hope to improve the response and success rates. Consequently, Soarce et al. [123] and Wu et al. [124] reported that the uptake and microdistribution of BPA-F in HN or glioma cancer patients could be increased by pulsed ultrasound suggesting that it should be evaluated clinically as a possible option for BNCT treatment [123,125,126].

Recently, Jai-Cheng reported the comparison of dose distributions in gross tumor volume between BNCT alone and BNCT combined with intensity modulation radiation therapy (IMRT) for head and neck cancer. He suggested that compared to single-fraction BNCT, the multi-fraction IMRT combined with single-fraction BNCT improves the treatment conformity and homogeneity and possibly local tumor control, especially for tumor whose volume is greater than 100 cm [127]. A recent study, published in Radiotherapy and Oncology, demonstrated the efficiency of BNCT in the treatment of locally recurrent head and neck squamous cell carcinoma (HNSCC) patients and also the factors that are favorable for the treatment response and survival [128]. This study, comprising 79 patients with locally recurrent HNSCC, who were treated with BNCT in Finland, between February 2003 and January 2012.

Some exciting findings of this study, highlighted by Ying Sun [129], are as follows:

1) Most patients with local recurrent HNSCC responded to BNCT.
2) A high minimum dose delivered to the tumor was a key predictive factor for treatment response, and the number of BNCT treatments was a minimally important factor for progression-free survival and overall survival.
3) Tumor size < 25 cm^3^ was found to be a favorable prognostic factor for survival and achieving complete response.
4) The minimum dose to the gross tumor volume was associated with the survival rates [128,129]. This was the first study to inspect the treatment outcomes in locally recurrent HNSCC patients in association with tumor dose from BNCT. However, this is a survey study. Thus, some key statistics on critical factors, such as adverse effects related to BNCT, human papilloma virus infection, and treatment-related deaths, were not measured or recorded, and could not control the consequences. This study delivers imperative evidence-based grounds for originating random clinical trials for the comparison of BNCT efficacy with other radiotherapies. To improve patient survival, this type of study is required to determine alternative therapies.

### 5.2. Cutaneous and Genital Cancer Treatment with BNCT

**Figure 10:**
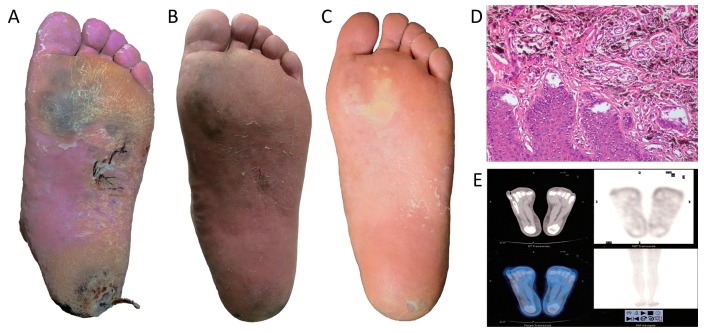
Gross examination of the skin lesions in the patient’s left foot 2 weeks (**A**), 5 weeks (**B**) and 24 months (**C**) after BNCT; (**D**) Pathological analysis after BNCT; (**E**) PET/CT scan after BNCT (reprint from Ref. [122]).

Another category of tumors that were treated by BNCT is cutaneous melanomas. Zhang et al. [130] reported the clinical treatment of cutaneous melanomas in three Chinese patients by using compact In-Hospital Neutron Irradiator (IHNI), which is specially fabricated and designed for BNCT. Out of these three patients, one has acral melanoma on the sole of his foot who had declined surgery (as indicated in Figure 10).

Yong et al. [122] reported that grade-II acute radiation injury was observed during the first four weeks after BNCT and was healed after treatment. No late radiation injury was found during the 24-month follow-up. The patient showed a complete response to BNCT, based on pathological analysis and gross examination, positron emission tomography-computed tomography (PET/CT) scan, indicating that BNCT is a potent treatment against malignant melanoma.

Two other patients, one with multiple metastatic cutaneous nodules on the right leg and the other with acral lentiginpus subungal melanoma of the right thumb, showed partial response [122] as per the Japanese clinical results in treating the cutaneous melanomas patients using BNCT. Hiratsuka et al. [60] reported four patients with genital malignancies, who were treated with BNCT and the patients included three with genital extramammary Paget’s disease (EMPD) and one patient with vulvar melanoma (VM). They underwent BNCT at the Kyoto University Research Reactor Institute (KURRI) facility between 2005 and 2014 using *para*-boronophenylalanine (*para-*BPA) as the boron delivery agent and were irradiated with an epithermal neutron beam between the curative tumor dose and the tolerable skin/mucosal doses. All patients showed similar tumor and normal tissue responses following BNCT and achieved complete responses within 6 months, and there were no recurrences observed during the follow-up periods ranging from 1.6–6.9 years.

The tumors of vulva and penis are very difficult to treat since these tumors are very poorly reactive to conventional photon irradiation and the surgery can be very harmful. As the treating EMPD and VM with BNCT in the above study of four patients resulted in complete local tumor control. Therefore, treating a large number of patients is required before making any definitive statement. The possible combination of new immunotherapeutic tactics [131,132] with BNCT would provide a better foundation for the treatment of cancers in difficult anatomic regions, like vulva, EMPD of the penis and scrotum with BNCT [60].

Lung cancer incidence is increasing worldwide and it’s considered to be the leading cause of cancer mortality. The treatment methods of lung cancer include surgery, chemotherapy and radiotherapy [133,134]. Among these, radiotherapy plays a progressively more important role in lung cancer treatment [135]. In recent years, many researchers proposed the use of BNCT for the treatment of lung cancer because of its significant superiority to conventional radiotherapies.

In its initial stages, the Kyoto University Reseach Team in 2006, performed a dosimetric study on malignant pleural mesothelioma to examine the feasibility of BNCT and confirmed that BNCT offered hope as a treatment modality for these patients [136]. After two years, they did clinical trials by treating a patient with mesothelioma and obtained good outcomes. The patient’s chest pain disappeared without late side effects [137]. In 2014, further studies by Farias, confirmed the effectiveness and feasibility of BNCT for treating lung cancer patients [138].

## 6. Secondary Cancer risk with BNCT

While performing BNCT, the dose deposited in the tumor, as well as the dose not fully excreted from the healthy organs, could cause secondary cancer. The secondary cancer risk cannot be ignored for cancer survivors, while paying attention to the side effects caused by radiotherapy, especially for younger people expecting to have longer life. Recently, the healthy organs of the younger brain tumor patients, treated with BNCT for secondary cancer risk, were investigated by Xinxin Zhang [139]. The high cancer incidence organs in China were considered and the same dose amount were obtained by tumor and organs using the Monte Carlo method and were radiated by computational phantom with Chinese physiological characteristics [140]. The effects of tumor depth (3-6 cm), irradiation geometry (RLAT/TOP/PA), patients age (10-15 years) and gender (male and female) on the secondary cancer risk were explored. Their results suggest that PA geometry showed the lowest secondary risk of cancer, while TOP show the highest, because under TOP geometry, most of the organs are directly exposed to the neutron beam and the equivalent doses are higher. It has been suggested that the secondary cancer risk in healthy organs would decrease with the age in both male and female patients, which might be related to the shorter life expectancy of the older patients. The higher secondary cancer risk in most organs of female patients than male patients of the same age were related to the higher β values in the LAR formula in female patients than male patients, or can be related to the thickness and height of the patient bodies. For the clinical application of BNCT, their findings provide reference about the secondary cancer risk, that requires further exploration. Hopefully, future research will identify new and better boron delivery agents for clinical use [87,141]. Randomized clinical trials help advance cancer therapy. Few randomized trials have been conducted on BNCT until now. Cooperative research groups are required to best accomplish more randomized clinical trials of BNCT.

## 7. Conclusions

No inherent limitations have been reported for introducing boron into pharmaceuticals. Only a few boron-containing drugs are available on the market. It is predicted that more boron-based drugs will be explored based on the development in nanotechnology, boron chemistry and newer installations of neutron resources in Asia. In this short review, some of the possible boron-based drugs were discussed, including those currently used in clinical trials.

The BNCT could serve as a promising therapy for malignant tumors, however the only clinically used boron delivery agents BPA and BSH have moderate selectivity. This encourages the search for new boron-based delivery agents. This review summarizes some of the recently reported boron delivery agents utilized with BNCT for in vivo and/or in vitro efficacy in theraupatic area. However, there are several critical issues, as itemized below, that must be addressed before applying BNCT as a useful modality for cancers treatment.

1. More effective boron-containing agents are required so that they can be used alone or in combination with other agents to deliver the necessary amount of boron to cancer cells.

2. The delivery of boron-containing agents to the cancer cells and cellular microdistribution must be optimized to improve its uptake, especially to diverse subpopulation of cancer cells.

3. Methods to provide semi-quantitative estimates of boron content in the residual cancer cells are essential.

4. For safety and efficacy of BNCT, the evaluation of randomized clinical trials are mandatory.

## Figures and Tables

**Table 1:** Summary of recently reported boron-based delivery agents in BNCT.

Materials	In Cell/In Tumor Model	Observation	Ref.
^10^BN	normal (HEK-293) and cancer (HeLa, MCF-7) cells	The promising antitumor effect was observed in HeLa cells. The thermal neutron fluence of ~6.3 × 10^12^/cm^2^ resulted in almost 50% cell killing of BN treated HeLa cells	[93]
BNNTs	B16-melanoma cell	The accumulation of BNNTs in B16-melanoma cell was three times higher than BSH and their antitumor effect with BNCT was also observed higher than BSH.	[94]
BNNTs-FA@PE	Hep G2 cells	Exhibited stronger cytotoxicity than free PE and BNNTs@PE complexes. Also, excellent antiproliferative activities in time- and dose-dependent manners. Additionally, it induced apoptosis of Hep G2 cells by reducing the mitochondrial membrane potential, activating Caspase-9 and Caspase-3.	[102]
PTL coated BNNPs	Triple negative breast cancer in mice	The coated BNNPs showed high boron accumulation in the tumor while maintaining its good ratio between tumor and nontumor cells. Suppression of tumor growth was observed with almost negligible side effects. Even these BNNPs shows high accumulation of B atoms inside the cells but they have failure in the therapeutic window for BNCT.	[46]
B-AuNP, 61-B-AuNP, ^123^I-B-AuNP, and ^123^I-61-B-AuNP	HER2	The The micro CT/SPECT imaging show the T/M ratio, assessed by ICP-MS, of mice injected by B-AuNP, 61-B-AuNP, ^123^I-B-AuNP, and ^123^I-61-B-AuNP was 4.91 ± 2.75, 41.05 ± 11.15, 1.91 ± 0.17, and 12.02 ± 0.94 respectively, at 12 h post-injection. The results show the successfully developed detectable HER2-targeting boron-containing AuNPs with high RCP and an acceptable yield.	[105]
(NH_2_NH_3_)^+^[7-NH_2_(CH_2_)_3_S- 7,8-C_2_B_9_H_11_]^-^ in Fluorescent GNCs dispersed in PBS	Cancer tumor in mice model	The accurate tumor imaging by EPR effect and long-term accumulation in tumor cell by nanometer size effect have been observed. This strategy is good for attaining the accurate position of tumor cells with CB and thus decreases the chances of normal tissue damage. In addition, it facilitates the real-time fluorescent visualization to monitor delivery process of the carborane to the targeted tumor, thus endorsing the effect of BNCT treatment through imaging guided therapy.	[106]
Gold NPs with PEG, functionalized with bis(dicarbollide), radiolabeled with ^124^I	*in vivo* with a mouse model	The results showed a poor accumulation in tumor and major accumulation in liver, lungs and spleen suggesting that the tuning the size and geometry of the gold core is essential	[109]
[^18^F]FBY PET	B16-F10 tumor	showed up to 6 % ID/g in B16-F10 tumor and notably low normal tissue uptake (tumor/muscle = 3.16 ± 0.48; tumor/blood = 3.13 ± 0.50; tumor/brain = 14.25 ± 1.54). Moreover, the administration of [^18^F]FBY tracer along with a therapeutic dose of FBY showed high accumulation in B16-F10 tumor and low normal tissue uptake.	[111]
iRGD-Modified Polymeric Nanoparticles	A549 tumor-bearing mice	Boron concentration ratios for tumor:normal tissue (tumor:muscle = 19.49, tumor:blood = 14.11) in A549 tumor-bearing mice was observed after 24 h of injection. The highest tumor accumulation of DOX was confirmed from both quantitative measurement and fluorescence imaging at 24 hrs after injecting iRGD-modified polymers	[114]
